# Let's get to work

**DOI:** 10.1017/gmh.2014.1

**Published:** 2014-07-04

**Authors:** G. Belkin

*Global Mental Health* (*GMH*) appears as a new Open Access journal, just as ‘Global Mental Health’ emerges as a new field. It is necessary, and timely: current international survey data suggest that mental illness is a key driver of population wellbeing, with evidence suggesting that mental healthcare might be a more cost-effective investment than physical healthcare for improving that outcome (Helliwell *et al.* 2013). The huge and growing morbidity burden attributable to common mental conditions (Murray *et al.* 2012; Vos *et al.* 2012), and their multiplier effect on the morbidity and mortality of other medical conditions (Prince *et al.* 2007), have prompted governments in low- and middle-income countries (LMICs) to increasingly and more aggressively pursue scalable solutions to close mental health treatment gaps (Ssebunnya *et al.* 2012; http://www.guardian.co.uk/global-development/2011/aug/29/ghana-new-mental-health-bill).

Mental health has increasingly become a part of the global health scientific agenda (Becker & Kleinman 2013). Among the signs of this are multiple special series on global mental health in *The Lancet*, a global Delphi exercise identifying research priority ‘Grand Challenges’ for global mental health reported in *Nature* (Horton 2007; Collins *et al.* 2011), a Grand Challenges Canada funding initiative to support those priorities (http://www.grandchallenges.ca/grand-challenges/gc4-non-communicable-diseases/mentalhealth/), a series of annual meetings to promote and cultivate global mental health research strategies and research capacity sponsored by the US National Institute of Mental Health, and endorsement of a Global Mental Health Action Plan by the World Health Assembly (WHO 2013). In addition, early work on Sustainable Development Goals (SDGs), expected to replace the Millennium Development Goals in 2015 as the set of global benchmarks driving much of the world's development investments, appears likely to include specific mental health targets and metrics for population wellbeing and happiness (Leadership Council of the Sustainable Development Solutions Network).

So it is especially timely to be launching a scientific and research journal specific to *Global Mental Health.* But such a launch carries a responsibility we intend to take seriously: to be a resource for helping establish just what this new ‘field’ is, and how successful it can be. Making the compelling case *for* mental health is no longer enough; we need clear and globally aligning metrics and targets, delivery designs, shared technical tools and language to *do* mental health, packaged in ways that are readily understandable to mainstream public health and social policy, planning, and healthcare delivery. We can mobilize the scientific field of global mental health to accelerate an ambitious scale of global investment, action, and organization for mental healthcare, in the same way the globalized fields of HIV care and maternal and child health (MCH) transformed the availability of effective and scaled care for those conditions.

To do this, the field needs to evolve so that it is not a niche field, but includes a broad array of expertise, including those of economists, organizational design and systems analysts, primary care providers and managers, quality improvement specialists, social media engineers, educators, users and advocates, and others beyond the usual ‘mental health’ disciplines itself.

This range of expertise will especially play a key role in developing the cross-sector strategies needed to realize the potential social and health gains accrued from comprehensively understanding and improving population mental health. Common mental disorders have begun to matter as a global priority because of the deep and broad impact their morbidity poses to health, social, and economic outcomes. Innovative, adaptable but scalable, designs for delivery of care therefore need to not only close gaps for treatment, but to do so in ways that optimize the reach of care to indeed have deep and broad impact – such as through intentional cross-sector task-shifting of skills and interventions ‘close-to-clients’ – by at the same time serving as platforms for executing preventive, early interventions and showing social impact. And the global perspective, the analytic point of view that can come from the sheer range of globalized comparative experience, is important in and of itself. A global perspective, changes perspective. It can aid more robust implementation and more robust science: such as broadened and globally representative sampling and methodologically diverse understandings of treatment and intervention efficacy, as well as of the etiology and epidemiological geography and behavior of mental illness, and mental health.

So the field, this new and still evolving scientific discipline of global mental health, is challenged to live up to these complex and broadly scoped ambitions and possibilities for the work of achieving global mental health. This journal was put together mindful of its potential to give shape and gather substance for that understanding of this discipline. Similarly, our approach to the scope of subject matter that we will consider relates to ‘mental health’, or ‘mental illness’, is broad, capturing the range of inputs and outcomes that has been powerfully described elsewhere as ‘Mental Capital’ – the range of intellectual and cognitive capabilities, interpersonal, social, and emotional repertoires, skills, and resiliencies, and morbidities that result from and contribute to the disordered mood, anxiety, substance use, and behavior that need to be addressed by mental healthcare, and need to be front and center to any effort to optimize and coherently talk about human and social development (Adler & Snibbe 2003; Hall & Lamont 2009; Shonkoff *et al.* 2009). *GMH* will apply the lens of a global perspective to building block implementation and clinical science areas across the range of Mental Capital, and we have categorized contributions and assigned the focus of Associate Editors along these areas – Soraya Seedat of the University of Stellenbosch to focus on contributions relating to questions of Etiology, Abdul Ghafar, Executive Director of the Alliance for Health Policy and Systems Research at WHO, to focus on Health Systems and Policy, Judith Bass of Johns Hopkins University to take on research contributions in the area of Interventions, and Helen Verdeli of Columbia University to manage topical areas of Teaching and Learning as it applies to the healthcare workforce, as well as all those receiving care from it (OECD 2013).

We will also take on the task of affirmatively seeking a broader range of contributors to this field in addition to the work of those mental health specialists, clinicians, and researchers who have built the field to date. We will signal to economists, social scientists, education, primary care and public health experts, how they can and need to also shape it. And in addition to publishing original research, the journal seeks to be a visible forum for more explicit and real-time discussion, such as through concept papers and reviews on questions and challenges facing the field. We will also make efforts specifically to support LMIC-based authors.

Some examples of how we plan to achieve these goals include taking steps such as forming a small Senior Advisory group, most of whom are recognized overall global health leaders, and leveraging their perspectives and networks, to, among other things, purposefully bring to bear involvement of an array of expertise on key issues in global mental health (Irene Agyepong, Ministry of Health, Ghana, Sustainable Solutions Development Network Health Co-Chair; Nigel Crisp, House of Lords, Former Executive Director NHS and Minister of Health, UK; Jed Friedman, Senior Economist, World Bank; Arthur Kleinman, Harvard College Professor, and Director Asia Center, Harvard University; Atif Rahman, Professor of Child Psychiatry, University of Liverpool; Hiro Yoshikawa, Professor of Globalization and Education, New York University). Convening that group has already borne fruit by bringing together an important range of contributors to our initial themed issue. Creating regular theme issues built around a call for papers as well as an expert working policy document or analysis is part of the strategy of *GMH* to broaden the field and deepen the connection between research and policy. The first such issue will be on Child Mental Health which will include a call for papers that cover the scope of Mental Capital http://journals.cambridge.org/images/fileUpload/documents/Call_for_papers_-_Child_Mental_Health3.pdf, but will also include a strategic white paper convened by *GMH* that will be authored by a group of recognized thought leaders in global child health, survival and nutrition, as well as child mental health more specifically. We have also designated a category of submissions that includes reports on work in progress, concepts and theory for this field, opinion pieces as well as qualitative research. And a specific Associate Editor role, taken on by Florence Baingana of Makerere University, has been identified to focus on cultivating LMIC-based networks of research and practice, and identifying potential new contributors from them. Finally, we are launching *GMH* as an Open Access title, to enable and promote unrestricted access to this research throughout the world, and for all articles accepted for publication in *GMH* during 2014 and 2015, Cambridge University Press will waive the article processing charge for all authors.

We would like to hear your thoughts about our goals, and along the way as to how we are doing. Please share with me directly your thoughts at globalmentalhealth@cambridge.org, and submit potential contributions through journals.cambridge.org/gmh/ifc. Global mental health has arrived. We will do our part at *GMH* to have it thrive.
